# Stiffness comparison of mushroom and straight SS and TMA lingual archwires

**DOI:** 10.1186/s40510-016-0140-2

**Published:** 2016-09-12

**Authors:** Luca Lombardo, Antonella Carlucci, Mario Palone, Francesco Mollica, Giuseppe Siciliani

**Affiliations:** 1 Postgraduate School of Orthodontics, University of Ferrara, Via Montebello 31, 44100 Ferrara, Italy; 2 Department of Engineering, University of Ferrara, Via Montebello 31, 44100 Ferrara, Italy

**Keywords:** Mushroom archform, Lingual straight wire, Stiffness, Stainless steel, TMA

## Abstract

**Background:**

The aim of this study is to investigate the relative stiffness of straight and mushroom lingual archwires of different diameters, cross sections and alloys, plotting their load/deflection graphs and using a modified three-point bending test.

**Methods:**

Fujita’s mushroom archwires and straight lingual archwires of different diameters, cross sections and alloys were derived by a virtual set-up of an equal malocclusion and were cut at their straight distal portion. These distal portions were tested using a modified three-point bending test by an Instron 4467 dynamometer and the forces, were exerted at 1-mm deflection and were compared on each resulting load/deflection curve by means of ANOVA (*p* < 0.05).

**Results:**

All upper lingual mushroom wires exerted significantly lower forces than the straight wire. Lower mushroom archwires were stiffer than their upper counterparts, which were longer and featured inset bends. In the lower arch, similar levels of forces were recorded for the two types of wire. Load-deflection curves were higher for the straight wires, and stiffness increased proportionally with their diameter.

**Conclusions:**

The stiffness of an archwire is a function of its diameter, length and the alloy it is made from. In lower lingual wires, there is little difference in stiffness between mushroom and straight wires, but in upper wires, the straight version is considerably stiffer. The greater bearing effect exhibited by the straight wire in the working and finishing phases makes it less susceptible to bowing effect and therefore preferable for sliding mechanics during en masse retraction, particularly in the upper arch.

## Background

Nowadays, lingual orthodontics is considered a suitable and valid method of correcting various types of malocclusion. Fujita’s mushroom-archwire technique, introduced in the 1970s, is effective [1, 2], but entails difficult clinical management, as it usually requires vertical and horizontal inset bends between the canine and premolar, which makes the outcome less predictable and comfortable [3]. However, in 1995, the lingual orthodontics was revolutionised by Scuzzo and Takemoto’s lingual straight wire [3].

As this technique evolved, new brackets with a lower profile (with shorter mesiodistal diameters and a thinner bracket pad) began to appear on the market, alongside new prescriptions and new straight wires (SW) designed for the lingual archform, squarer than the rounder version launched in the 1990s. These innovations improved the reliability and speed of lingual orthodontics, not to mention patient comfort during the treatment [4]. Indeed, one of the most important advantages of using the lingual straight wire technique is that sliding mechanics enable extraction spaces to be closed without the need for difficult modelling of the closing loop used in non-frictional extraction space closure [3, 5].

Lingual orthodontics is generally used in adult patients who want to improve their appearance without repercussions on their social life. Because the possibility of arch expansion in these patients is limited, clinicians must often opt for an extractive treatment and full canine retraction followed by en masse retraction of the six anterior teeth as a single unit [6–8], to avoid unsightly space between the lateral incisor and canine during the treatment. This, however, requires greater orthodontic forces and consequently increases the likelihood of side effects and loss of anchorage.

As a matter of fact, it is advisable in frictional space closure to use lighter forces and heavier wires with a stiffness able to counteract the negative effect of elastic chain forces [6, 7] reducing the vertical and horizontal bowing effect and providing good finishing and optimal space closure. As the stiffness of a wire is inversely proportional to its length [8–10], it has been hypothesised that a straight lingual archwire, being shorter than the corresponding mushroom archwire, should aid the clinician in keeping bowing effect under control [11–13].

We set out to test this hypothesis by investigating the relative stiffness of different mushroom and straight-form lingual archwires for both arches in vitro, using a modified three-point bending test and plotting their load/deflection graphs. Many previous studies have investigated the relative wire stiffness comparing lingual and labial archwires, respectively [9, 14], but they have tended to focus on interbracket distance and its correlation with archwire stiffness during the initial alignment and levelling phase, whereas we decided to focus on the final stage of lingual orthodontic therapy, namely extraction space closure with sliding mechanics.

To our knowledge, no study has yet been conducted with the aim of comparing the stiffness of two different lingual archforms used for en masse retraction in extraction space closure. We therefore set out to test the above hypothesis, with a view to confirm the suitability of the lingual straight wire system for space closure mechanics and to identify the most suitable working lingual archwire as regards shape, size and alloy.

## Methods

**Fig. 1:**
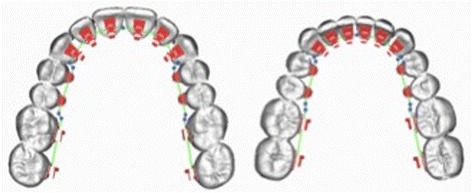
Digital set-up of an ideal patient with moderate crowding using a lingual mushroom archwire (UMW and LMW)

**Fig. 2:**
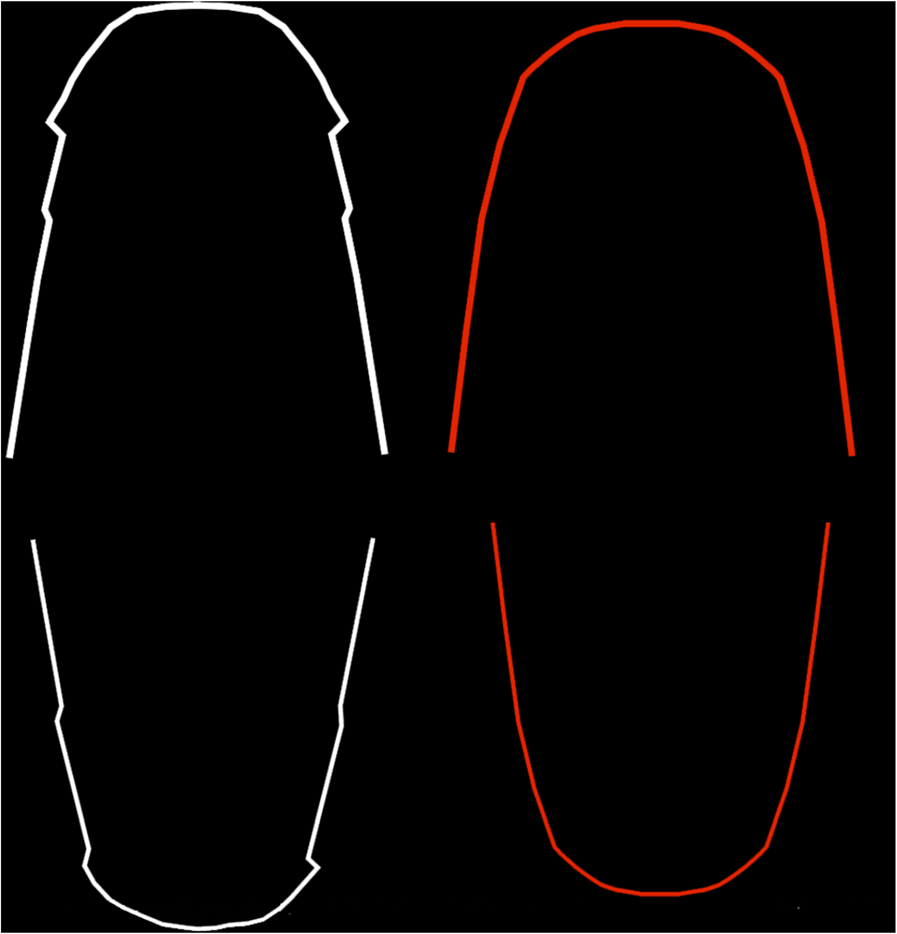
Lingual mushroom and lingual straight archwires are generated by the same 3D virtual set up both for maxillary and mandibular arches

A 3D virtual model set-up (Orapix system), based on a single ideal patient with moderate crowding, was generated and used to design one set of mushroom archwires—upper (UMW) and lower (LMW)—and one set of straight wires (SW)—upper (USW) and lower (LSW)—according to the Scuzzo-Takemoto method (Figs. 1 and 2).

ImageJ software was used to measure the length of all archwires, and the exact respective differences in length between the straight and the mushroom wires of both arches were calculated as a percentage proportion. We used these virtual measurements to cut the real archwires to the same lengths at their straightest distal portion, and the percentage proportion calculated was used as a basis to obtain the real length of the mushroom archwire samples including their inset bends. Five types of sample wire were tested in this study, namely 0.016 × 0.016-in. SS, 0.016 × 0.022-in. SS, 0.018 × 0.018-in. SS, 0.018 × 0.025-in. SS and 0.0175 × 0.0175-in. β-Ti. The archwires were provided by Ormco (Orange, CA, USA) (Table. 1).

**Table 1:** Lingual archwires tested in the study cut at their distal straightest portion according to percentage proportion length calculated by virtual set-up

Archwire size	Commercial name	Lot number
SS 0.016 × 0.016	Ormco	206-0005
SS 0.016 × 0.022	Ormco	206-0006
TMA 0.017 × 0.017	Ormco	202-0018
SS 0.018 × 0.018	Ormco	204-2302
SS 0.018 × 0.025	Ormco	206-0008

Each sample was mounted as an edgewise wire in four vestibular passive self-ligating brackets (slot 0.022 × 0.028 in.; Damon 3Mx, Ormco) that had been glued to an acrylic resin base in such a way as to create a 16-mm span between the internal sides of two adjacent brackets.

The study followed the ISO 15.841 guideline to perform orthodontic test [15]. So each wire was subjected to a three-point bending test, modified to simulate clinical conditions as accurately as possible [16]. An Instron 4467 dynamometer (Instron, Norwood, Mass) connected to a 100-N load cell was used to regulate the force applied, and archwire deflection was achieved via a metal blade, with a curvature range of 1 mm at its extremity, fixed to the load cell.

**Fig. 3:**
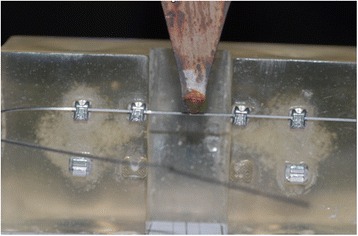
Deflection, with 1 mm blade, of the portion of a real archwire using a modified three binding test (ISO 15.841 guideline)

**Fig. 4:**
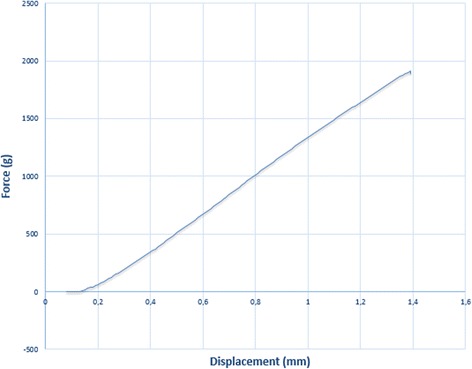
Example of a Load/Deflection curve of a 0.018 X 0.018 SS lingual straight archwire

Each wire was deflected 1 mm, at a deflection speed of 1 mm/min (Fig. 3). To obtain reliable data, we tested each wire three times. The bending stiffness of each wire was then determined by plotting a force/deflection graph and calculating the slope of the linear portion of the curve (Fig. 4). Data were collected by means of a personal computer connected to the measuring device and processed using Labview 8.5. The data were presented in spreadsheet form using Microsoft Excel (Microsoft Corporation, Redmond, Wash), and a load/deflection graph, showing deflection of the test strip on the *x*-axis and the force exerted on the *y*-axis, was plotted for each sample of each type of wire tested. Each curve was taken as representative of the loading phase and indicative of the force exerted on the teeth during orthodontic treatment. All tests were performed under dry conditions and at room temperature of 20± 2 °C.

In order to verify a normal distribution of the data, a Kolgomorov-Smirnov (KS) test was applied. The level of significance was set at 0.05.

Statistical analysis was aimed to evaluate the stiffness of each type of wire (SW, UMW and LMW), using one-way analysis of variance (ANOVA) to evaluate the effects of each type of wire on the forces exerted during the experiment. Descriptive statistics, including means and standard deviations, were calculated for each type of wire alloy.

## Results

The Kolgomorov-Smirnov test revealed that a normal distribution of the data is always respected (*p* > 0.05) (Table. 2).

**Table 2:** *D* value of the non-parametric Kolgomorov-Smirnov test for each type of lingual archwires and their statistical significance (*p* < 0.05*)

Archwire size	*D* value
SS 0.016 × 0.016	0.20 (NS)
SS 0.016 × 0.022	0.15 (NS)
TMA 0.017 × 0.017	0.31 (NS)
SS 0.018 × 0.018	0.22 (NS)
SS 0.018 × 0.025	0.36 (NS)

*NS* not significant

The virtual set-up yielded an USW +11.58 % shorter than the UMW and a LSW +3 % shorter than the LMW.

**Fig. 5:**
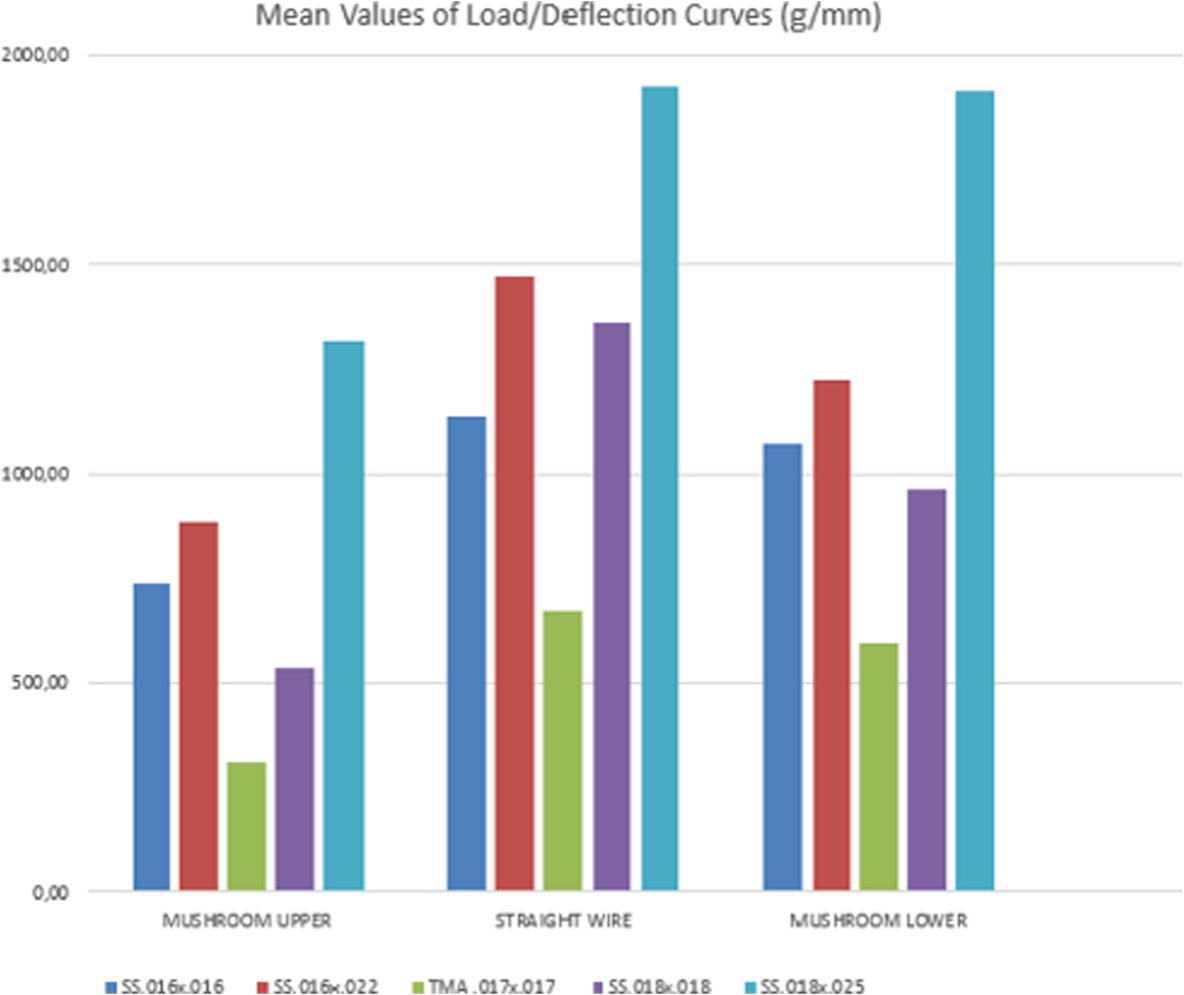
Mean values of Load/Deflection curves of SS 0.016 × 0.016-inch, SS 0.016 × 0.022-inch, SS 0.018 × 0.018-inch, SS 0.018 × 0.025-inch and β-Ti 0.0175 × 0.0175-inch archwires using a modified three bending test according to ISO 15.841 guideline
- ¥ The difference between UMWs and USWs in the force exerted at the same amount of deflection was statistically significant, but the difference between LMWs and LSWs did not always reach statistical significance (Table 3).
- ¥ All SWs and MWs showed a tendency for the force exerted to increase with increasing archwire diameter.
- ¥ Titanium molybdenum alloy (TMA) wires are much softer than stainless steel wires (SS) and therefore exert far less force.

A comparison of the forces exerted by straight and mushroom archwires showed that:

- ¥ For a given deflection, straight archwires exerted greater force with respect to mushroom wires (UMW and LMW) of the same section and alloy (Fig. 5).

As expected, each type of UMW generated significantly lower forces than the SW counterpart, due to the inset bend, which increases the length of the wire and therefore its elasticity (Fig. 5). There was a greater similarity between the LSW and LMW load/deflection curves, presumably owing to the minimal difference in length between the two (about 3 %). Likewise, statistical analysis of these data via ANOVA *F* test revealed significant differences between the stiffness of USWs and UMWs, for all the couple of wires tested (Table 1). Indeed, the average stiffness of the SS 0.016 × 016 SW was 35 % greater than the SS 0.016 × 016 UMW, while it was only 6 % greater than SS 0.016 × 016 LMW. Moreover, the average stiffness of the SS 0.018 × 0.018 SW was 60 % greater than the SS 0.018 × 0.018 UMW, but it was only 4 % greater than SS 0.018 × 0.018 LMW. Similarly, the 018 × 025 SS SW exerted a greater force than the UMW (*p* < 0.001) but very similar force with respect to the LMW (*p* = 0.17).

**Table 3:** Differences in main values of load/deflection curves (g/mm) of each archwire size and their statistical significance using *F* ANOVA test (*F* < 0.001*; *F* < 0.01**; *F* < 0.05***)

	Differences in main values of load/deflection curves (g/mm)	
SS 0.016 × 0.016	400*	69
SS 0.016 × 0.022	585**	246***
TMA 0.017 × 0.017	363.4*	78***
SS 0.018 × 0.018	828.9**	399.3***
SS 0.018 × 0.025	605*	5
	Straight wire vs mushroom upper	Straight wire vs mushroom lower

Further statistically significant differences were found between the SS 0.016 × 0.022 USW, and the SS 0.016 × 0.022 UMW (*p* < 0.01), and the SS 0.016 × 0.022 LSW and the SS 0.016 × 0.022 LMW (*p* < 0.05). Similarly, 018 × 0.018 SS showed that SW developed greater forces than both UMW (*p* < 0.01) and LMW (*p* < 0.05). Hence, while a strong statistical significance was found for differences between USWs and UMWs in all cases, in the comparison of LSWs and LMWs, it was only reached in archwires of SS 0.016 × 0.022 in., SS 0.018 × 0.018 in. and TMA 0.175 × 0.0175 in. and to a lesser extent (*p* < 0.05) with respect to maxillary lingual archwires. Comparison of wire alloys indicated that TMA wires produce lesser forces and are softer than SS wires.

## Discussion

Orthodontic lingual treatment is a feasible way to treat several malocclusion especially in adult patients, who are most likely to seek an aesthetic solution [17]. Lingual straight wire method simplified orthodontic mechanisms and increases the predictability of orthodontic outcomes, and the impact of this revolution on lingual orthodontics was comparable to that brought about by the introduction of Andrew’s labial straight wire technique in 1972 [18].

Scuzzo and Takemoto realised that if they cut the clinical crowns off a plaster cast, the buccolingual distances at the gingival margin did not vary substantially between canine and first premolar. This led them to conclude that lingual straight wire method was a feasible way if the brackets were placed as close to the gingival margin as possible. Thus, they identified the lingual straight plane (L-S plane), the optimal plane on which to place vertical bracket slots [5].

This method made possible a frictional space closure with sliding mechanism to correct arch length discrepancy, anteroposterior jaw relationship and to improve the soft-tissue profile [6, 7, 19].

A key factor during this type of treatment, especially during a frictional en masse retraction, is good anchorage control. This will prevent pre-contacts between the maxillary lingual brackets and the mandibular incisors that would otherwise induce posterior dysfunction and inhibit retraction. Moreover, posterior segments tend to tip mesially, leading to lateral open bite at the premolars and loss of lateral function. This phenomenon is known as the vertical bowing effect and can occur when the stiffness of the archwire is overcome by the active forces exerted by the power chains. This can also cause distobuccal molar and mesiobuccal canine rotation and arch expansion at the premolars, causing the so-called transverse bowing effect [6, 7].

Above-mentioned side effects could be counteract modulating elastic chains forces to an optimum level and by using straight lingual archwires with suitable stiffness. We set out the test to prove the hypothesis that lingual straight wire are most suitable than mushroom lingual archwires to take under control these side effects thanks to their major stiffness due to their minor length by a modified three bending test performed through an Instron 4467 dynamometer (Instron, Norwood, Mass).

The recommended lingual archwires for partial canine and en masse retraction of the six anterior teeth are 0.016-in. SS and 0.016 × 0.022-in. SS in the mandibular arch, whereas stiffer wires are recommended for the maxillary arch, specifically 0.017 × 0.025 SS and 0.0175 × 0.0175 TMA, which should enable better control of maxillary incisor torque [19, 20, 21].

In this respect, according to our measurements, all straight wire samples were stiffer than their mushroom counterparts. Although we acknowledge the limitations of conducting an in vitro rather than in vivo study, we also show that the difference in stiffness between the two archwire shapes is particularly relevant in the maxilla, where there is a greater difference in their lengths (about 11.5 %). Indeed, the forces exerted by the UMWs was far lower than that measured for the corresponding USWs, whereas the relative forces exerted by the LMWs and the corresponding LSWs were more similar, and statistically significant differences were not seen in all cases. This could be an advantage in the lower arch with some kinds of archwires, but not all. Indeed, the TMA samples were deflected to a far greater extent than the stainless steel samples, in accordance with the literature, which states that the modulus of elasticity (*E*) of TMA is about 30 % with respect to that of stainless steel, and TMA wires therefore exert lower forces at the same amount of deflection [12, 13, 22].

The literature also states that the force released by an archwire increases proportionally with its diameter but decreased proportionally with its length. Hence, the stiffness of an archwire depends on both its modulus of elasticity (*E*), i.e. the alloy, and geometric factors, its second moment of inertia (*I*). For archwires with a rectangular cross section, *I* is h^3^w/12, whereas for those with a round cross section, it is Π*r*^4^/64 [11, 12, 22, 23].

In a maximum anchorage case, when sliding mechanics are used, better anchorage control in the posterior segment can be achieved with a stiffer wire. To this end, it is interesting to note how a straight 0.016 × 0.016-in. SS for the upper arch provides 35 % more stiffness with respect to a mushroom wire. When 0.018 × 0.018-in. SS SWs and UMWs were compared, this difference in stiffness rose to 60 %. As regards the type of alloy, we confirm the findings that TMA wires are not rigid enough to counteract the elastic forces exerted by power chains and should therefore be reserved for non-frictional space closure rather than sliding mechanics. Moreover, microscopic analysis of TMA wires has revealed a very rough surface with the worst coefficient of friction of any of the orthodontic archwires, in which also makes them unsuitable for use in frictional extractive space closure [22]. As a stiff wire provides greater control of the system, minimising vertical and transversal bowing and the other unwanted effects mentioned above [6, 7], it follows that the straight wire would seem to be preferable to the mushroom wire in this respect, particularly in maxillary arch. Incidentally, mushroom wires are also plagued by the difficult management of bending in the non-friction extractive space closure due to the small inter-bracket distances and the greater tendency of adult patients to experience irritation of the soft tissues provoked by loops [24, 25]. Concerning the archwire cross section, it is preferable to choose an archwire with a cross-sectional area enough large to guarantee the stability of the system, but not so large as to increase friction at the wire/bracket interface.

## Conclusions

Despite the study emphasises some known facts and proven hypothesis like correlation of the stiffness archwire with its cross section (rectangular or round), its dimension and its alloy, our research shows that:

- Lingual straight archwires should be preferred during frictional space closure by virtue of their major stiffness, capable to take under control bow side effects. Moreover, they also simplified the job of orthodontic practitioners, making arch coordination less difficult and simpler sliding mechanics possible, as well as reducing chair-side time [6, 26].
- Lingual straight archwires should be preferred specially in the maxillary arch where we registered an important statistical differences for all the couple of wires compared.
- LMWs generally develop forces almost equal to LSWs, but there were statistically and clinically significant differences (*p* < 0.05) in three of the archwire types tested (SS 0.018 × 0.022, SS 0.016 × 0.016 and TMA 0.175 × 0.0.175) but to a lesser extent. Therefore, the decision to perform frictional space closure in the mandibular arch is less critical with respect to maxillary arch.

Although the inset bends in mushroom archwires have always made it difficult to determine their stiffness, our study appears to show the greater predictability of lingual straight wires in terms of good extraction space closure through sliding mechanisms. This aids the clinician to counteract the side effects of a mechanical closure, that is to say vertical and transversal bowing effects, better preserving the shape of the working archwire. The differences between mushroom and straight lingual archwires seem to be less significant in the lower jaw, where arch length is more comparable, but, in general, it can be stated that stainless steel archwires are the most suitable for non-frictional space closure in extractive orthodontic treatment.

## Endnotes

This study is an in vitro study, so it is not a representative of a complex biological system like the mouth where muscular activity, saliva and individual responses to orthodontic movements could not be simulated. Moreover, we performed the test with passive self-ligating vestibular brackets rather than lingual brackets in order to avoid the frictional resistance caused by steel or elastomeric ligatures and each error linked to operator skill in performing ligatures.

## Abbreviations

UMW: Upper lingual Muschroom Wire
LMW: Lower lingual Muschroom Wire
USW: Upper lingual Straight wire
LSW: Lower lingual Straight wire
